# *In vivo* functional brain mapping in a conditional mouse model of human tauopathy (*tau*_p301l_) reveals reduced neural activity in memory formation structures

**DOI:** 10.1186/1750-1326-8-9

**Published:** 2013-02-04

**Authors:** Pablo D Perez, Gabrielle Hall, Tetsuya Kimura, Yan Ren, Rachel M Bailey, Jada Lewis, Marcelo Febo, Naruhiko Sahara

**Affiliations:** 1 Department of Psychiatry, University of Florida McKnight Brain Institute, Gainesville, Florida, 32610, USA; 2 Center for Translational Research in Neurodegenerative Disease and Department of Neuroscience, University of Florida, Gainesville, FL, 32610, USA; 3 Department of Aging Neurobiology, National Center for Geriatrics and Gerontology, Obu-shi, Aichi, 474-8511, Japan

**Keywords:** Tauopathy, Neurodegenerative disease, Alzheimer’s disease, rTg4510, Manganese enhanced MRI

## Abstract

**Background:**

Tauopathies are characterized by intracellular deposition of the microtubule-associated protein tau as filamentous aggregates. The rTg4510 mouse conditionally expresses mutant human tau protein in various forebrain areas under the Tet-off expression system. Mice develop neurofibrillary tangles, with significant neuronal loss and cognitive deficits by 6 months of age. Previous behavioral and biochemical work has linked the expression and aggregates of mutant tau to functional impairments. The present work used manganese-enhanced magnetic resonance imaging (MEMRI) to investigate basal levels of brain activity in the rTg4510 and control mice.

**Results:**

Our results show an unmistakable curtailment of neural activity in the amygdala and hippocampus, two regions known for their role in memory formation, but not the cortex, cerebellum, striatum and hypothalamus in tau expressing mice.

**Conclusion:**

Behavioral impairments associated with changes in activity in these areas may correspond to age progressive mutant tau_P301L_-induced neurodegeneration.

## Background

Accumulation of intracellular neurofibrillary tangles (NFTs) consisting of microtubule-associated protein tau is a major hallmark of Alzheimer’s disease (AD) and related neurodegenerative diseases regarded as ‘tauopathies’ [1-3]. Findings of tau mutations in subjects affected by frontotemporal dementia and mutant tau expression systems, which lead to tau-positive inclusions, neuron loss and behavioral abnormalities in various animal models, have established a role of this protein in neurodegeneration [4-11].

The rTg4510 mouse line was specifically developed to model aspects of human tauopathy, with overexpression of human tau containing the P301L mutation that is associated with frontotemporal dementia with parkinsonism linked to chromosome 17 (FTDP-17-Tau). Expression of human tau in the rTg4510 mouse is controlled by the tetracycline transactivator (tTA) transgene under the Ca^2+^/calmodulin-dependent protein kinase IIα (CaMKIIα) promoter. This leads to selective tau expression from an otherwise transcriptionally inactive tau transgene [12]. Mice develop robust intracellular deposition of tau protein in cortico-limbic areas, which is physiologically relevant to AD and other tauopathies, and they also show age-related forebrain atrophy.

The main pathological features of rTg4510 mice have been obtained as a result of postmortem analysis of brain tissue, except recent work employing multiphoton microscopical analysis [13,14]. The advent of rodent magnetic resonance imaging methods can fill the need for investigating *in vivo* correlates of early signs of disease. Recent developments in the animal imaging have led to the use of the manganese ion (Mn^2+^) as a neuronal contrast agent that provides a useful tool for functional brain mapping at good spatial resolution [15,16]. MEMRI thus provides a noninvasive approach for mapping neural activity in transgenic mice. Mn^2+^ is highly paramagnetic and enhances signal intensity in T_1_ weighted brain images [17,18]. It enters neurons largely through voltage dependent calcium channels, and possibly also via vesicular reuptake mechanisms, which correlates well with changes in synaptic firing of neuronal populations [19-21]. Other MEMRI studies in rodents have shown Mn^2+^ signal intensity in various ROI, including the hippocampus, under basal conditions [17,18]. Here, we performed *in vivo* brain MEMRI of rTg4510 mice and non-transgenic (nonTg) littermates. Mutant rTg4510 mice sustain significant tau-associated hippocampal atrophy at 6 months of age and above and this was anticipated to result in less Mn^2+^ signal within this region in basal un-stimulated conditions. We observed clear differences in basal neural activity between mutant tau expressing mice and controls, with the magnitude of activity being lower in rTg4510 mice. Our study extends previous applications of MEMRI in the study of axonal transport rates in transgenic mice by showing reduced neural activity in two structures involved in learning and memory [22-24].

## Results

### Methodological considerations

**Figure 1:**
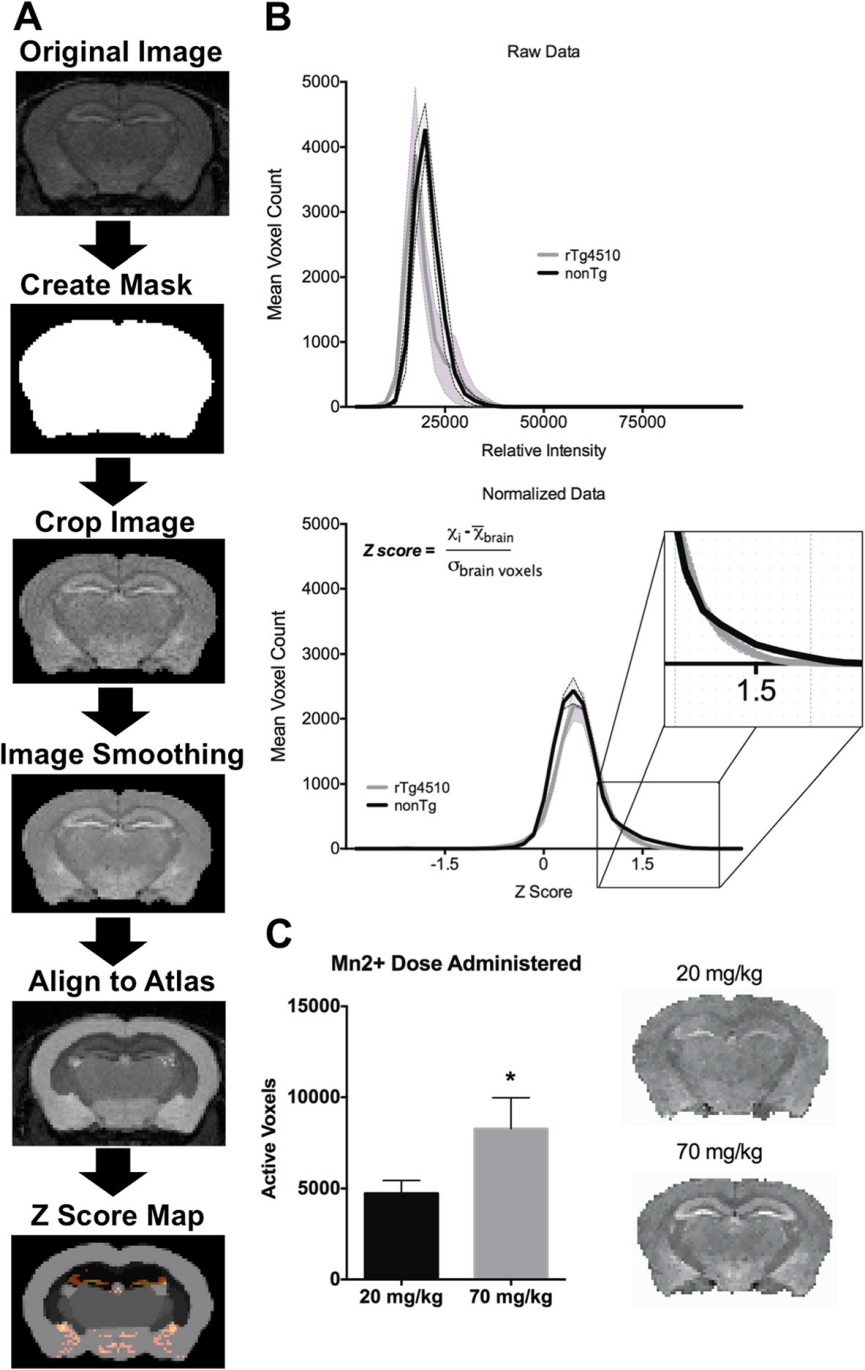
**Summary of manganese (Mn^2+^) enhanced MRI (MEMRI) preprocessing and analysis procedure. A**) Steps in image processing and generation of Z score maps. **B**) Intensity histograms for raw data (top panel) and data presented as normalized z scores (bottom panel). A comparison is made between control nonTg mice (n = 5; dark line) and rTg4510 mice (n = 5; grey lines). Data presented as mean voxel counts (± standard error). Voxel-wise Z score calculation equation used is shown. Inset in bottom graph highlights upper portion of the histogram accounting for voxels with increased signal intensity (Z ≥ 1). **C**) Effect of Mn^2+^ dose on mouse brain signal intensity in control scans normalized using the Z score procedure. Data presented as mean number of voxels at Z scores equal or above 1 (n=6 for 20 mg/kg MnCl_2_ administration, n=5 for 70 mg/kg MnCl_2_ administration, respectively). Coronal brain maps to the right of the graph are both at a set signal intensity threshold between Z =−2 and Z = +3. *p = 0.001 unpaired two-tailed t-test.

We closely analyzed the intensity histograms of rTg4510 and control mice (histograms in Figure 1B). The probability of finding voxels with a Z score of 1 or more was less than 5% (*p* < 0.05; Figure 1B bottom graph inset). This corresponds to 7.9% of the total population of brain voxels for rTg4510 mice and 10.6% for nonTg mice (unpaired t-test t_8_=3.4 *p* = 0.01). About 0.05% of voxels had values of Z = −1 or less (*p* < 0.0003); thus, negative Z values were negligible and not of interest in the present analysis. Only voxels with positive Z values were considered in subsequent ROI analyses. We also carried out a series of pilot experiments to determine the optimal dose of Mn^2+^. Comparison of the two doses, shown in Figure 1C, highlights a dose-dependent effect on signal intensity. Control nonTg mice (n = 6) administered 20 mg/kg Mn^2+^ showed signal increases of significantly smaller magnitudes than a separate cohort of nonTg mice (n = 5) administered 70 mg/kg (t-test t_9_=4.757 *p* = 0.001). The higher dose is just below previous doses reported in the literature for mouse (88 mg/kg in [18]), however, in our hands 70 mg/kg did not result in any overt motor or other visible disturbances in nonTg and rTg4510 mice.

### ROI analysis

**Figure 2:**
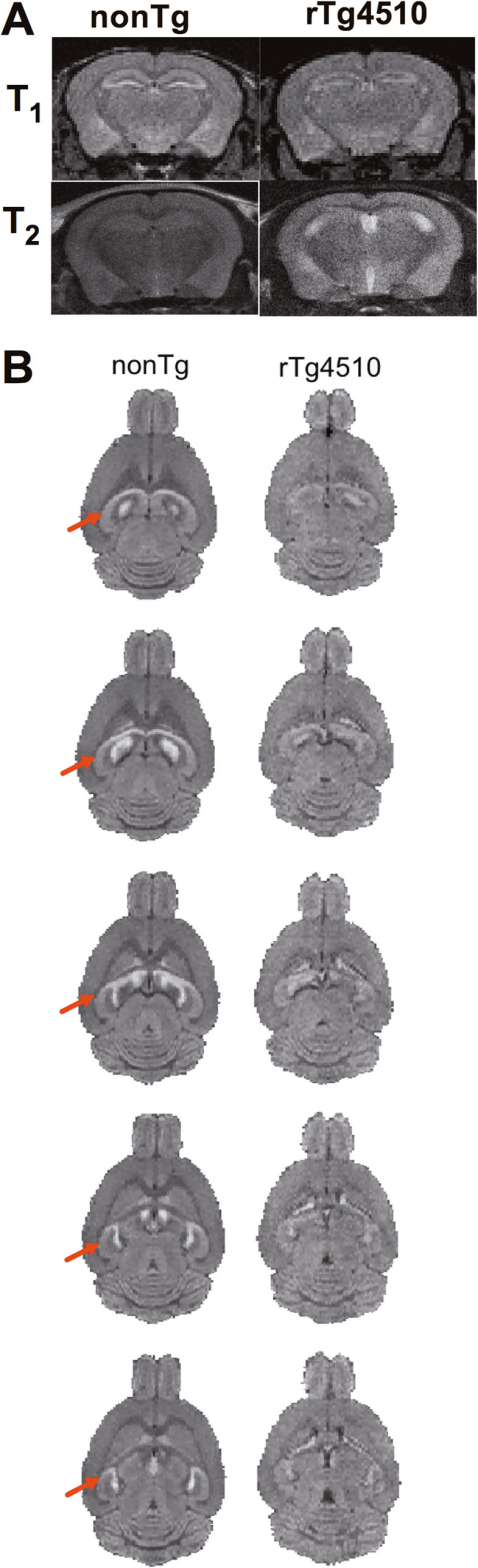
**Functional mapping of basal neural activity in control nonTg and mutant tau expressing rTg4510 mice using MEMRI. A**) Coronal view of dorsal hippocampal signal intensity in nonTg and rTg4510 mice. **B**) Axial sections illustrating from top-to-bottom (dorsal-to-ventral) slices the signal intensity within the dorsal and ventral hippocampal subareas (red arrows).

**Figure 3:**
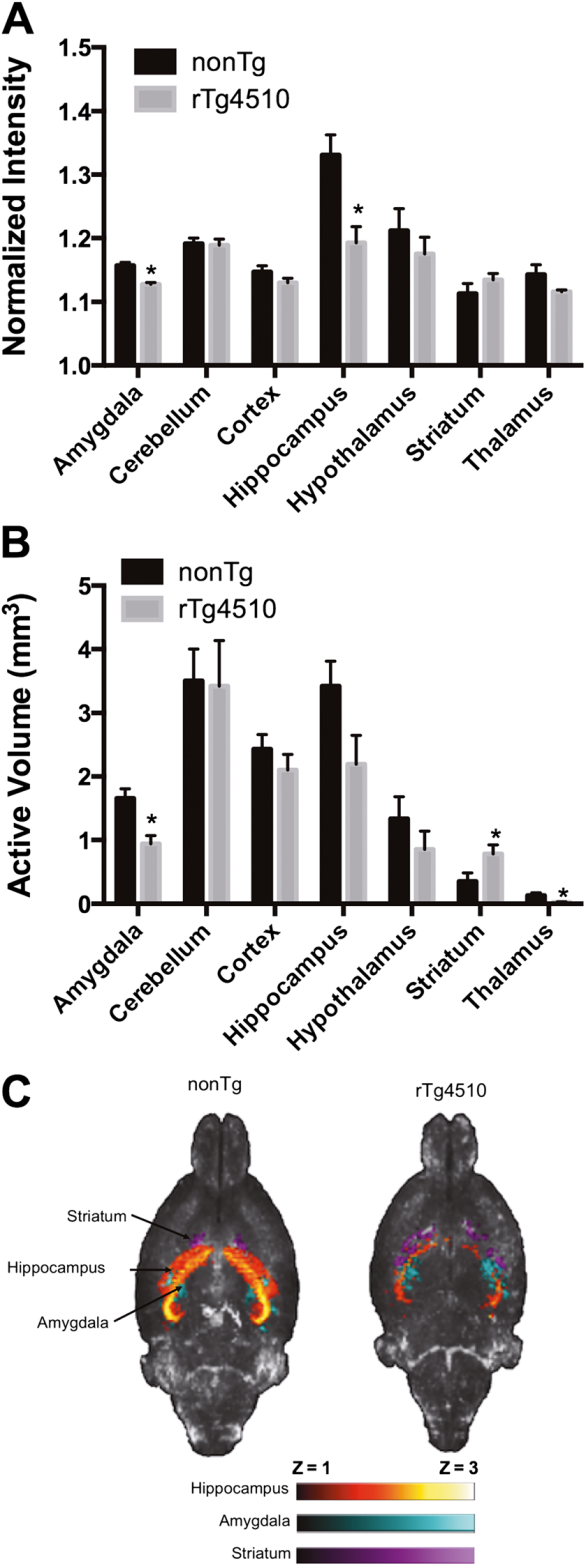
**MEMRI of control nonTg and mutant tau expressing rTg4510 mice. A**) Normalize signal intensity across various ROI (expressed as mean Z scores of voxels equal or above 1). **B**) Active volume across various ROI (expressed as mean volume – in mm^3^ - of voxels equal or above 1). **C**) Mean intensity projection images highlighting intensity and active volume in areas of the striatum, hippocampus and amygdala (purple, yellow-orange and light blue, respectively). *p < 0.05 t-test comparing means of nonTg vs rTg4510 mice.

High signal intensity levels were observed in subregions of the hippocampus, hypothalamic areas, central amygdala, lateral habenula, basal forebrain area, layers of the olfactory bulb, and cerebellum, as previously reported for systemically administered Mn^2+^[18]. We noted also that there was high Mn^2+^ associated signal in the amygdala, mostly pronounced in the central amygdala area. Further qualitative inspection of each of the MRI scans indicated a smaller hippocampal volume in rTg4510 mice than control mice (Figure 2A T_2_ weighted images), consistent with previous imaging of rTg4510 mice [25]. Normalized voxel intensity values (in Z scores), and the number of voxels with an intensity value equal to or above the threshold value (mean signal and activated volume, respectively) were extracted from each ROI. Signal intensity increases were more pronounced in dorsal areas of the hippocampus, particularly the dentate gyrus (Figure 2B). However, increased signal intensity was still observed in ventral CA3 (Figure 2B). Signal intensity was noticeably lower in the hippocampus of rTg4510 mice than in controls (Figure 2B). Generalized signal intensity differences between groups did not explain this difference, as these were normalized and values rescaled to within a similar range (Figure 1). In addition, cortical volume, which is reportedly reduced in 5-month old rTg4510 mice [25], did not show similar reductions in neural activity (Figure 3). Thus, the reduced signal intensity is selective for hippocampus and amygdala, of rTg4510 mice. Significant reductions in signal intensity were observed in amygdala (t_8_=5.0 *p* = 0.001) and hippocampus (t_8_=3.407, *p* = 0.009) of rTg4510 mice (Figure 3A). We also analyzed the number of voxels exceeding a threshold Z score value of 1 (presumed “active regions” during basal conditions) (Figure 3B). As stated in the methods section, we normalized the images and set a threshold at 1 standard deviation above the mean. Thus, all voxels below this threshold were set to zero and signal intensities above the threshold were compared. The active volume was lower in rTg4510 mice than in control nonTg mice in amygdala (t_8_=3.7, *p* = 0.006) and thalamus (t_8_=2.5, *p* = 0.04). The hippocampus showed the same trend as with signal intensity (t_8_=2.0, *p* = 0.07). Interestingly, the striatum showed a greater active volume in rTg4510 mice than controls (t_8_=2.2, *p* = 0.05). Mean intensity projection images in Figure 3C illustrate the main findings. Hippocampal and amygdala signal intensity is lower in rTg4510 mice than controls. The striatum shows the opposite effect with greater active volume in rTg4510 mice.

**Figure 4:**
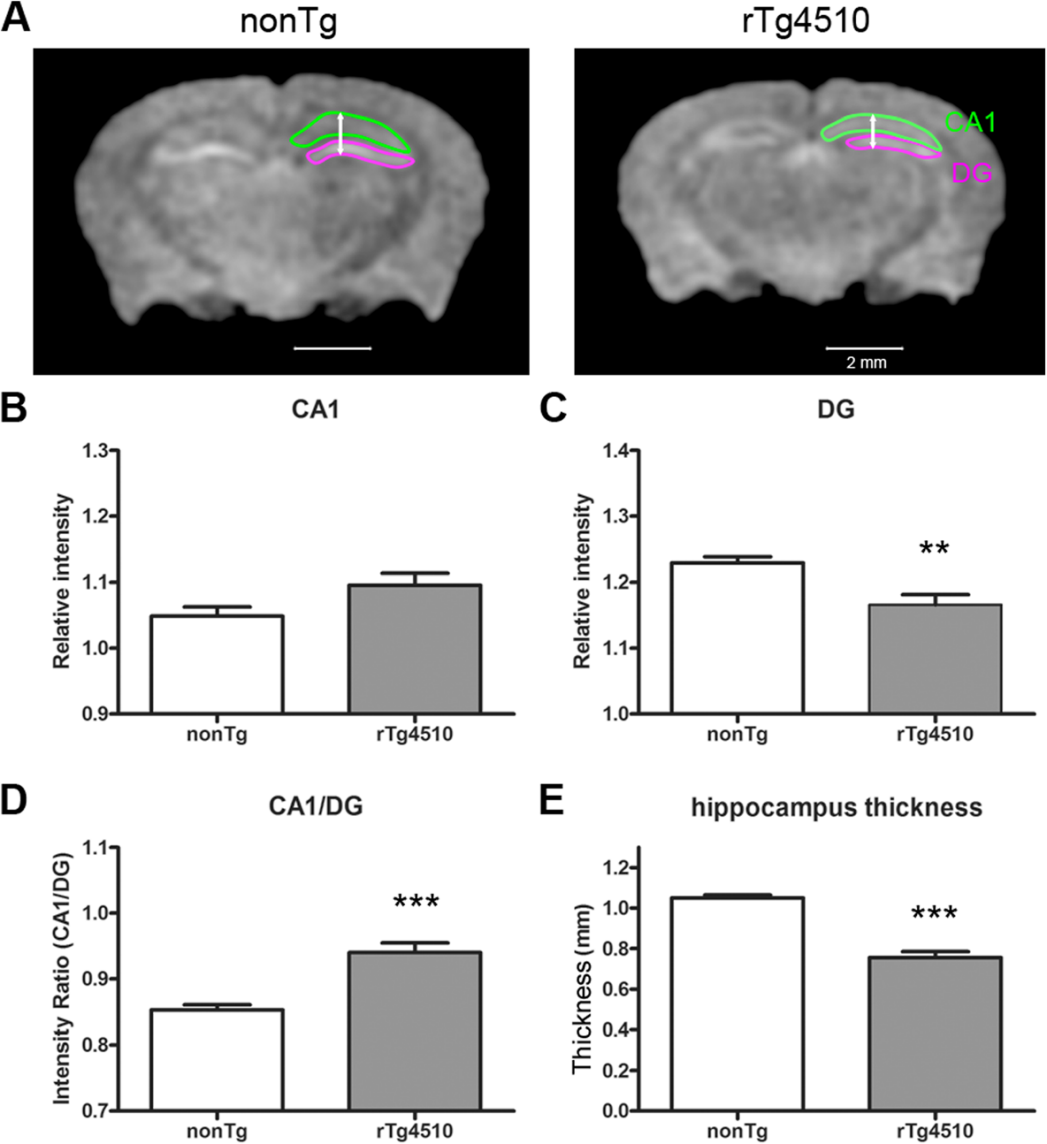
**Manganese enhanced intensity and thickness of hippocampus. A**) Representative coronal sections of non Tg and rtg4510 mouse brains. The coronal slices were at the level Bregma −2 mm. Sub-regions of hippocampus were drawn as CA1 (green) and DG (purple). Arrows showed the analyzed position of hippocampus thickness. The position was set at 1.5 mm from center. Scale bar=2 mm. **B**) Relative intensity of CA1 region was normalized by averaged intensity of whole brain. **C**) Relative intensity of DG region was normalized by averaged intensity of whole brain. **D**) Ratio of intensity between CA1 and DG. E) Comparison of hippocampus thickness between non Tg (n=5) and rTg4510 (n=5) mice. **p<0.01, ***p<0.001.

**Figure 5:**
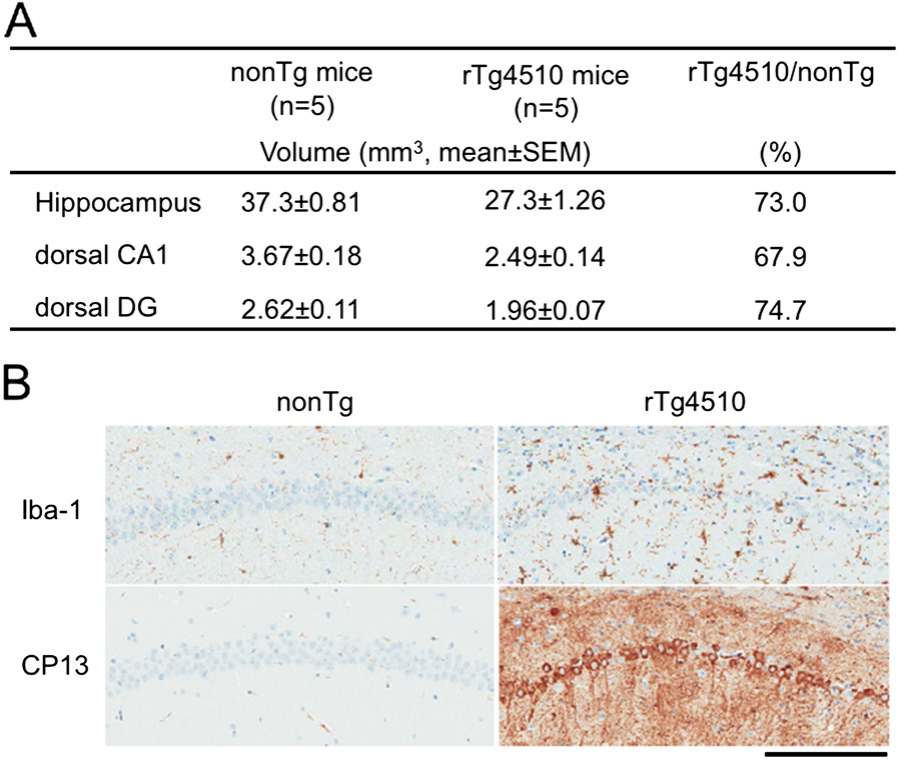
**Volumes and immunohistochemical images of hippocampus sub regions. A**) Hippocampal volumes of segmented *in vivo* images. Whole hippocampus, dorsal CA1 and dorsal dentate gyrus regions (DG) were manually segmented. Ratios between nonTg and rTg4510 were shown as percentage (%). **B**) Iba-1 and CP13 antibody stainings of hippocampus (CA1) in 6 month-old rTg4510 and nonTg littermate. Activated microgila cells detected by Iba-1 were intensely stained in the rTg4510 hippocampus. CP13 staining in rTg4510 showed hyperphosphorylated tau accumulation in CA1 neurons. Scale bar=200 mm.

Given the expression levels of human mutant tau in the hippocampal formation of this mouse model, we further analyzed this region for sub region specific signal intensity and volumetric differences (Figure 4A). As indicated above, anatomical differences were clearly discerned in scanned images of rTg4510 and nonTg mice (Figure 2A). Surprisingly, it was noted that differences in signal intensity in the hippocampus was not uniformly reduced throughout in rTg4510 mice. There was a greater signal intensity in the hippocampal dorsal CA1 region in rTg4510 mice (Figure 4B), which greatly contrasted with the differences observed at the level of the dentate gyrus (DG; Figure 4C). When analyzed as intensity ratio between CA1 and DG, there is significantly greater differential activity in rTg4510 compared to nonTg mice (Figure 4D). This was observed despite reduced hippocampal thickness in these animals (Figure 4E). Reduced volume was further confirmed by manual segmentation of dorsal hippocampal sub regions. Approximately 30% of hippocampus volume was decreased in rTg4510 compared to nonTg mice (Figure 5A). On the other hand, enlarged microglia staining was clearly visible despite significant neuronal loss in CA1 region in 6 month-old rTg4510 mice (Figure 5B).

## Discussion

rTg4510 mice develop NFTs and show significant loss of hippocampal neurons at around 5–6 months. Neuronal loss may be severe (~80%) by 8 months [26]. These factors are likely to contribute to impairments observed in spatial navigation memory previously reported using the water maze test [27], a behavior that is associated with neuronal activity in the dorsal hippocampus. The present study used *in vivo* MEMRI to map brain activation in control and rTg4510 mice under baseline, non-stimulated conditions. We observed significantly lower signal intensity, which may reflect underlying reductions in neuronal firing in rTg4510 versus control littermates. This effect appeared selective for memory formation structures, such as the hippocampus and amygdala, and was not observed in other ROI such as the septum, cerebellum, striatum, midbrain and brainstem. The hippocampus and amygdala play important roles in learning and memory, with the hippocampal structures contributing significantly to declarative memory while the amygdala contributes to implicit or emotional memory formation. While there is growing evidence that progressive reductions in hippocampal activity lead to generalized memory impairments, the implications for the amygdala are new for the rTg4510 mouse. The amygdala, particularly the basolateral regions, develop NFT neuropathology, however, a mechanism in rTg4510 mice remains to be thoroughly investigated. Dickey et al. (2009) immunohistochemically stained for tau and found no staining in thalamus. Indirect effects of tau pathology (or tau expression) in other regions, such as the neocortex, may have contributed to the differences observed here. In addition, we observed increases in signal intensity (activity) in the striatum, an effect that might reflect some changes in disinhibitory process arising from cortical inputs. However, despite the lack of any evidence of neuronal loss in the striatum, this region does accumulate NFTs beginning around 5.5 months [26].

Progression of NFT pathology in rTg4510 mice has been considerably explored by several groups. Immunohistochemical analysis revealed that rTg4510 mice develop pre-tangle pathology at 2.5 M and fully formed NFTs at 4 M in the cortex and at 5.5 M in the hippocampus [12,28]. NFT pathology in the hippocampal formation occurs in a distinctly staged sub regional pattern beginning from CA1 to CA3 and dentate gyrus [28]. Biochemical analysis of the rTg4510 hippocampus confirmed that the progression of NFT formation was drastically increased from 4 months to 6 months of age [27,29-31]. In agreement with these neuropathological observations, our *in vivo* MRI analysis from rTg4510 mice (5.5-6.3 M) showed significant reduction of both neural activity and volume in the hippocampus. We observed significant reduction of signal intensity in the dentate gyrus of rTg4510 mice. Because CA1 pyramidal neurons were significantly decreased at this age, other sub regions of the hippocampus may receive less signaling associated with neural activity. Similar to our present observations, Yang et al. (2011) showed increased reactive astrocytic gliosis and enlarged microglia in CA1 from 8 M male rTg4510. One interesting possibility may be the sequestering of Mn^2+^ in microglial cells occurs in rTg4510 CA1. This would either increase CA1 signal or pull away signal from the DG. An in-depth neurochemical investigation is warranted. An important follow up will be to control the expression of transgenic tau and the progression of NFT formation and/or neuronal loss through the administration of doxycycline and examine whether mice show unbalanced neural activity across hippocampal sub regions at different stages of disease progression.

A series of experiments using MEMRI have been carried out to examine axonal transport rates along the olfactory and optic tracts in transgenic mice for amyloid precursor protein (APP) expression. Using a fast temporal series of T_1_ weighted image acquisitions it was shown that there is an age progressive decline in olfactory tract axonal transport rates of Mn^2+^ that worsened after plaque formation. In a subsequent study it was demonstrated that loss of APP in knockout mice resulted in reduced axonal transport rates for Mn^2+^, which was recovered by over-expressing human wildtype tau [22]. Similar deficits in axonal transport rates have been reported in regions of the visual system and hippocampal formation of APP knockout mice [24] and in the olfactory system of triple transgenic mice expressing APP and human presenilin [23]. The above references illustrate the versatility of the MEMRI method. Localized *in vivo* treatment can be used to track not only global brain activity as in the present work, but also transport rates on selective regions of the CNS [32]. Our present results are consistent with recent work published by Kimura et al. (2007) showing lower activity in hippocampal and surrounding parahippocampal areas of aged mice with hyperphosphorylated tau protein. Levels of signal intensity in the parahippocampal areas highly correlated with performance on a spatial learning task [33]. In the latter study Mn^2+^ was provided to animals only 4 hours prior to imaging session and thus provides evidence of significant brain uptake of Mn^2+^ across the blood brain barrier of mice. This is supported by *in vitro* studies showing rapid uptake of Mn^2+^ in brain, which appears to be aided by both active and passive transport processes [34].

To our knowledge, this is the first MEMRI study examining neural activation in the rTg4510 mouse. Previous work on this transgenic mouse has focused on chemical and volumetric differences compared to controls. Both male and female mutant tau-expressing mice show reduced total brain volume which is largely associated with lower cortical and hippocampal volume [25]. We observed this feature as well and also noted the previously reported widening of ventricles [25]. This is consistent with postmortem tissue analysis in these same animals [12,26]. Proton MR spectroscopy showed greater myoinositol concentrations in the hippocampus and thalamus of rTg4510 mice, and this is consistent with increased gliosis [25]. Interestingly, N-acetylaspartate, which is often taken as a marker of neuronal function, was not different between controls and rTg4510 mice. We observe here that *in vivo* neural activity is reduced during baseline conditions in these animals. The previously reported lower number of neurons [26], which may lead to lower hippocampal volume [25], also may have accounted for the generally lower signal intensity in this region in rTg4510 mice. However, cortical neural activity was not different between control mice and rTg4510 mice despite the reduced volume in the latter. This could signify that the lower neural activity is due to reduced functional activity in memory structures. We also found that the reduction in hippocampal signal intensity is not uniform through the subanatomical layers of this paleocortical region. rTg4510 mice show a somewhat modest, but significant increase in signal intensity in the CA1 region. It is tempting to speculate on what such an effect may be associated with. One possibility could be due to compensatory changes in neuronal activity across the dorsal hippocampal areas; however, this would potentially result in maintenance of higher levels of performance on spatial navigation and memory tasks. Past data do not support such a contention at this time. One alternative might be linked to gliosis, which would be consistent with past work by Yang et al. (2012). Microglial cells migrating to this region might sequester Mn^2+^ to such an extent that they result in increased signal intensity. This would not be due to increased neuronal activity, but rather it would be in line with the pathogenicity observed with tau over-expression at this age of the mice. This is somewhat supported by the literature. Quinolic acid lesions of neural tissue result in greater levels of Mn^2+^ accumulation than in controls, thus supporting internalization of Mn^2+^ in both neurons and glial cells [35]. In conclusion, we find that mice over expressing human mutant tau(_P301L_) show lower brain activity in selected regions involved in memory formation. The present novel findings are consistent with previous behavioral and biochemical studies in the rTg4510 mouse. Moreover, the methods used provide a novel approach to explore the specific relation between genetically-driven expression of pathogenic tau and functional changes over neurodegenerative disease progression.

## Methods

### Mice

The parental P301L tau responder line, parental tTA activator line, and the resultant F1 rTg4510 mice and littermates were generated and maintained as previously described [12]. Mice were maintained on a standard diet lacking doxycycline to ensure that transgenic tau was expressed throughout the lifetime of the experimental animals (weights were 25-40 g at the time of imaging). All mice were kept in standard size mouse cages (29 x 18 x 13 cm; up to 5 per same sex groups) at 20-26°C on a daily 12 hour light–dark cycle (light on during 0700 – 1900) with *ad libitum* access to food and water. Mice kept under these conditions were handled twice per week. Animals were generated and cared for in accordance with the guidelines published in the Guide for the Care and Use of Laboratory Animals (8^th^ Edition, 2011) and in adherence to the National Institutes of Health and the American Association for Laboratory Animal Science guidelines. All procedures involving live mice received prior approval from the Institutional Animal Care and Use Committee of the University of Florida.

### Manganese chloride pretreatment

Manganese (II) chloride tetrahydrate was purchased from Sigma-Aldrich Chemical Co. (St. Louis, MO, USA) and was dissolved in ddH_2_O and sterile filtered prior to administration. We first carried out pilot experiments to examine the optimal MnCl_2_ dose (20 and 70 mg/kg) that produced T_1_ weighted signal enhancement in the absence of motor impairments. These experiments were carried out on a separate cohort of animals at 5.3-6.3 months of age (M) and resulted in selection of the 70 mg/kg MnCl_2_ solution. This dose produced results similar to those reported in previous work [18]. In order to map basal brain activity in rTg4150 mice (n=5; 5.5-6.3 M) and control nonTg littermates (n=5; 5.5-6.3 M), MnCl_2_ was administered at a dose of 70 mg/kg, i.p. 24 hrs prior to MRI scanning. Animals were handled every two weeks and acclimatized to the investigator providing the injections. After injections, mice were returned to their home cage and imaged the following day.

### Magnetic resonance imaging

Images of anesthetized mice were collected on an actively shielded 4.7-Tesla Magnex Scientific MR scanner controlled by Agilent Technologies VnmrJ 3.1 console software. A 38-mm quadrature transmit/receive radiofrequency (RF) coil tuned to 200 MHz was used (Insight NeuroImaging Systems, LLC, Leominster, MA). Anesthesia was initially induced under 2.0-2.5% isoflurane (0.1 mL/min) delivered in 100% oxygen for 30–60 seconds and levels were then maintained between 1.0-1.25% throughout the entire setup and imaging session. Mice were placed prone on a custom-made plastic bed with a respiratory pad placed underneath the abdomen. They were covered with disposable paper towel to aid in preserving body warmth. Body temperatures were maintained using a warm air recirculation system that received feedback from a fiber optic thermocouple microprobe (SA Instruments, Inc., New York). Respiratory rates were monitored continuously and maintained between 20–30 beats per minute by adjusting isoflurane levels between 1–1.25%. Incisors were secured on the front end of the plastic bed to minimize motion. The front half of the bed was aligned and clamped inside the quad RF coil and placed inside the isocenter of the scanner. Images were acquired using a T_1_ -weighted 3D gradient echo sequence with the following parameters: repetition time (TR) = 35 ms, echo time (TE) = 12 ms, flip angle α = 50°, data matrix 256 X 128^2^ (along read X phase X slice directions), size 30 mm X 20 mm^2^ (resolution 200 X 156 μm^2^ along the same read X phase X slice directions). Total scan time per mouse was 57 minutes.

### Data processing and statistical analysis

Figure 1A provides a diagram describing portions of the image processing and data analysis procedure. Accumulation of Mn^2+^ within brain tissue shortens its T_1_ recovery time, leading to enhanced signal intensity [15,16]. However, there is significant scan-to-scan variability that requires normalization before carrying out between-groups’ statistics. Various normalization procedures have been used in the past such as normalizing to phantoms containing chemical constituents of grey matter [36], normalization to specific regions of interest (ROI) not anticipated to sequester Mn^2+^ during specific tasks or stimuli [32], or normalizing brain to muscle tissue near the sampled ROI [22]. In the present study, we used a voxel-wise Z-score normalization procedure similar to [37] that facilitates within and between groups comparisons at a set threshold level and minimizes some of the between scan variability (Figure 1B). Image processing was carried out using itk SNAP (http://www.itksnap.org), OsiriX (http://www.osirix-viewer.com/), and image math scripts available on FSL (http://www.fmrib.ox.ac.uk/fsl/). As shown in Figure 1A, a whole brain mask was generated per each scan and used to first crop out the non-brain portions of 3D MR scans (non-brain voxels were set to 0). A non-linear filter was applied to the cropped images to reduce noise without altering resolution [38]. Scans were aligned with a segmented atlas of the adult C57BL/6 J mouse brain (http://brainatlas.mbi.ufl.edu/) using an automated affine linear registration tool from FSL [39]. To overcome differences in brain volume between control and rTg4510 mice [28,40], subject-specific versions of the atlas were made based on alignment and warping of the atlas coordinates to each subjects’ scan space (12 parameter affine registration). Alignment was verified for accurate delineation of ROI and manually adjusted if needed (Figure 1A) The anatomical location of ROI was compared to a histological atlas of the mouse brain [41]. Finally, each scan was converted to a Z score map through a voxel-wise normalization procedure. The mean signal intensity across the entire extracted brain volume ($\bar{x}$) was subtracted from each voxel (*xi*) and then divided by the variance (σ):

$$\mathrm{ZScore}=\frac{\mathrm{xi}-\bar{x}}{\sigma }$$

A pre-set threshold of Z ≥ 1 was selected based on *a priori* observation of individual datasets and a close inspection of their intensity distribution histograms. This corresponds to a threshold of 1 standard deviation above the mean. All voxels with z score values below this threshold were set to zero. The intensity histograms in Figure 1B show mean voxel counts for the entire brain of 5 rTg4510 mice and 5 control mice included in the study. Rescaling of intensity values based on each subjects’ signal intensity variance allowed consistent sampling of brain voxels showing increase signal across each subject. The voxels exceeding the threshold value of z =1 were considered in our statistical analysis as having higher signal intensities (exceeding 1 standard deviation above the mean signal intensity across the whole brain). The segmented ROIs in the digital atlas of the mouse brain included the neocortex, striatum, globus pallidus, olfactory bulb, hypothalamus, septum/basalforebrain, midbrain, brainstem, thalamus, hippocampus, amygdala and cerebellum. Upon analyzing all regions, the hippocampus showed major differences between rTg4510 and control mice. However, the digital atlas does not segment sub regions of the hippocampus. Therefore, a separate analysis was also carried out to examine in detail the volume of the hippocampus, which has previously been reported to be smaller in rTg4510 [28]. For this analysis, dorsal hippocampal sub regions CA1 and DG were manually traced. Mean normalized signal intensity values for each ROI were compared using an unpaired two-tailed t-test (homoscedastic variances, α ≤ 0.05).

### Immunohistochemical staining

Formalin fixed brains were paraffin embedded and cut into sagittal (5 μm) sections. Standard immunohistochemical procedure was implemented with the Dako Universal Autostainer (Dako, Carpinteria, CA). Primary antibodies used CP13 (phosphorylated tau at Ser202, provided by Dr. P.Davies, Albert Einstein College of Medicine) and ionized calcium-binding adaptor molecule 1 (Iba-1, Wako Chemicals USA, Richmond, VA).

## Abbreviations

ROI: Region of interest; rTg4510: Conditional transgenic mouse for human mutant tau expression; Mn2+: Manganese chloride; MnCl2: Manganese chloride; NFT: Neurofibrillary tangles; CaMKIIa: Calcium/calmodulin kinase IIα; tTA: Tetracycline transactivator; AD: Alzheimer’s disease; nonTg: Non-transgenic mice; RF: Radiofrequency; DG: Dentate gyrus; MEMRI: Manganese enhanced magnetic resonance imaging; MRI: Magnetic resonance imaging.

## Competing interests

The authors have no conflict of interest to disclose

## Authors’ contributions

PDP, carried out the imaging data collection. GH, contributed to animal imaging and treatments. TK, contributed to data analysis and manuscript writing up. YR, carried out animal breeding, treatments and tissue processing. RB, carried out the immunohistochemistry, JL, contributed research animals manuscript editing and aided in conceptualization of study. MF, helped conceive the study, manuscript write up and did parts of the data analysis. NS, conceived study, manuscript write up and helped carry out portions of the study. All authors read and approved the final manuscript.

## References

[B1] Lee VM Goedert M Trojanowski JQ Neurodegenerative tauopathies Annu Rev Neurosci 2001 24 1121 1159 10.1146/annurev.neuro.24.1.1121 11520930

[B2] Swaab DF Dubelaar EJ Hofman MA Scherder EJ van Someren EJ Verwer RW Brain aging and Alzheimer’s disease; use it or lose it Prog Brain Res 2002 138 343 373 12432778 10.1016/S0079-6123(02)38086-5

[B3] Binder LI Guillozet-Bongaarts AL Garcia-Sierra F Berry RW Tau, tangles, and Alzheimer’s disease Biochim Biophys Acta 2005 1739 216 223 10.1016/j.bbadis.2004.08.014 15615640

[B4] Lewis J McGowan E Rockwood J Melrose H Nacharaju P Van Slegtenhorst M Gwinn-Hardy K Paul Murphy M Baker M Yu X Neurofibrillary tangles, amyotrophy and progressive motor disturbance in mice expressing mutant (P301L) tau protein Nat Genet 2000 25 402 405 10.1038/78078 10932182

[B5] Gotz J Chen F Barmettler R Nitsch RM Tau filament formation in transgenic mice expressing P301L tau J Biol Chem 2001 276 529 534 11013246 10.1074/jbc.M006531200

[B6] Tanemura K Akagi T Murayama M Kikuchi N Murayama O Hashikawa T Yoshiike Y Park JM Matsuda K Nakao S Formation of filamentous tau aggregations in transgenic mice expressing V337M human tau Neurobiol Dis 2001 8 1036 1045 10.1006/nbdi.2001.0439 11741399

[B7] Tatebayashi Y Miyasaka T Chui DH Akagi T Mishima K Iwasaki K Fujiwara M Tanemura K Murayama M Ishiguro K Tau filament formation and associative memory deficit in aged mice expressing mutant (R406W) human tau Proc Natl Acad Sci USA 2002 99 13896 13901 10.1073/pnas.202205599 12368474 PMC129794

[B8] Allen B Ingram E Takao M Smith MJ Jakes R Virdee K Yoshida H Holzer M Craxton M Emson PC Abundant tau filaments and nonapoptotic neurodegeneration in transgenic mice expressing human P301S tau protein J Neurosci 2002 22 9340 9351 12417659 10.1523/JNEUROSCI.22-21-09340.2002 PMC6758022

[B9] Schindowski K Bretteville A Leroy K Begard S Brion JP Hamdane M Buee L Alzheimer’s disease-like tau neuropathology leads to memory deficits and loss of functional synapses in a novel mutated tau transgenic mouse without any motor deficits Am J Pathol 2006 169 599 616 10.2353/ajpath.2006.060002 16877359 PMC1698785

[B10] Yoshiyama Y Higuchi M Zhang B Huang SM Iwata N Saido TC Maeda J Suhara T Trojanowski JQ Lee VM Synapse loss and microglial activation precede tangles in a P301S tauopathy mouse model Neuron 2007 53 337 351 10.1016/j.neuron.2007.01.010 17270732

[B11] Eckermann K Mocanu MM Khlistunova I Biernat J Nissen A Hofmann A Schonig K Bujard H Haemisch A Mandelkow E The beta-propensity of Tau determines aggregation and synaptic loss in inducible mouse models of tauopathy J Biol Chem 2007 282 31755 31765 10.1074/jbc.M705282200 17716969

[B12] Santacruz K Lewis J Spires T Paulson J Kotilinek L Ingelsson M Guimaraes A DeTure M Ramsden M McGowan E Tau suppression in a neurodegenerative mouse model improves memory function Science 2005 309 476 481 10.1126/science.1113694 16020737 PMC1574647

[B13] Spires-Jones TL de Calignon A Matsui T Zehr C Pitstick R Wu HY Osetek JD Jones PB Bacskai BJ Feany MB In vivo imaging reveals dissociation between caspase activation and acute neuronal death in tangle-bearing neurons J Neurosci 2008 28 862 867 18216194 10.1523/JNEUROSCI.3072-08.2008 PMC6670992

[B14] de Calignon A Fox LM Pitstick R Carlson GA Bacskai BJ Spires-Jones TL Hyman BT Caspase activation precedes and leads to tangles Nature 2010 464 1201 1204 10.1038/nature08890 20357768 PMC3091360

[B15] Pautler RG Silva AC Koretsky AP In vivo neuronal tract tracing using manganese-enhanced magnetic resonance imaging Magn Reson Med 1998 40 740 748 10.1002/mrm.1910400515 9797158

[B16] Aoki I Tanaka C Takegami T Ebisu T Umeda M Fukunaga M Fukuda K Silva AC Koretsky AP Naruse S Dynamic activity-induced manganese-dependent contrast magnetic resonance imaging (DAIM MRI) Magn Reson Med 2002 48 927 933 10.1002/mrm.10320 12465100

[B17] Pautler RG Mongeau R Jacobs RE In vivo trans-synaptic tract tracing from the murine striatum and amygdala utilizing manganese enhanced MRI (MEMRI) Magn Reson Med 2003 50 33 39 10.1002/mrm.10498 12815676

[B18] Lee JH Silva AC Merkle H Koretsky AP Manganese-enhanced magnetic resonance imaging of mouse brain after systemic administration of MnCl2: dose-dependent and temporal evolution of T1 contrast Magn Reson Med 2005 53 640 648 10.1002/mrm.20368 15723400

[B19] Aschner M Gannon M Manganese (Mn) transport across the rat blood–brain barrier: saturable and transferrin-dependent transport mechanisms Brain Res Bull 1994 33 345 349 10.1016/0361-9230(94)90204-6 8293318

[B20] Narita K Kawasaki F Kita H Mn and Mg influxes through Ca channels of motor nerve terminals are prevented by verapamil in frogs Brain Res 1990 510 289 295 10.1016/0006-8993(90)91379-U 2158851

[B21] Fukuda J Kawa K Permeation of manganese, cadmium, zinc, and beryllium through calcium channels of an insect muscle membrane Science 1977 196 309 311 10.1126/science.847472 847472

[B22] Smith KD Kallhoff V Zheng H Pautler RG In vivo axonal transport rates decrease in a mouse model of Alzheimer’s disease NeuroImage 2007 35 1401 1408 10.1016/j.neuroimage.2007.01.046 17369054 PMC2063432

[B23] Kim J Choi IY Michaelis ML Lee P Quantitative in vivo measurement of early axonal transport deficits in a triple transgenic mouse model of Alzheimer’s disease using manganese-enhanced MRI NeuroImage 2011 56 1286 1292 10.1016/j.neuroimage.2011.02.039 21338698 PMC3098472

[B24] Gallagher JJ Zhang X Ziomek GJ Jacobs RE Bearer EL Deficits in axonal transport in hippocampal-based circuitry and the visual pathway in APP knock-out animals witnessed by manganese enhanced MRI NeuroImage 2012 60 1856 1866 10.1016/j.neuroimage.2012.01.132 22500926 PMC3328142

[B25] Yang D Xie Z Stephenson D Morton D Hicks CD Brown TM Sriram R O’Neill S Raunig D Bocan T Volumetric MRI and MRS provide sensitive measures of Alzheimer’s disease neuropathology in inducible Tau transgenic mice (rTg4510) NeuroImage 2011 54 2652 2658 10.1016/j.neuroimage.2010.10.067 21035554

[B26] Spires TL Orne JD SantaCruz K Pitstick R Carlson GA Ashe KH Hyman BT Region-specific dissociation of neuronal loss and neurofibrillary pathology in a mouse model of tauopathy Am J Pathol 2006 168 1598 1607 10.2353/ajpath.2006.050840 16651626 PMC1606598

[B27] Berger Z Roder H Hanna A Carlson A Rangachari V Yue M Wszolek Z Ashe K Knight J Dickson D Accumulation of pathological tau species and memory loss in a conditional model of tauopathy J Neurosci 2007 27 3650 3662 10.1523/JNEUROSCI.0587-07.2007 17409229 PMC6672413

[B28] Ramsden M Kotilinek L Forster C Paulson J McGowan E SantaCruz K Guimaraes A Yue M Lewis J Carlson G Age-dependent neurofibrillary tangle formation, neuron loss, and memory impairment in a mouse model of human tauopathy (P301L) J Neurosci 2005 25 10637 10647 10.1523/JNEUROSCI.3279-05.2005 16291936 PMC6725849

[B29] Sahara N DeTure M Ren Y Ebrahim AS Kang D Knight J Volbracht C Pederson JT Dickson DW Yen SH Lewis J Characteristics of TBS-extractable hyperphosphorylated tau species: Aggregation intermediates in rTg4510 mouse brain J Alzheim Dis 2013 33 249 263 10.3233/JAD-2012-121093 PMC3514650 22941973

[B30] Barten DM Cadelina GW Hoque N DeCarr LB Guss VL Yang L Sankaranarayanan S Wes PD Flynn ME Meredith JE Tau transgenic mice as models for cerebrospinal fluid tau biomarkers J Alzheimers Dis 2011 24 2 127 141 21422517 10.3233/JAD-2011-110161

[B31] Dickey C Kraft C Jinwal U Koren J Johnson A Anderson L Lebson L Lee D Dickson D de Silva R Aging analysis reveals slowed tau turnover and enhanced stress response in a mouse model of tauopathy Am J Pathol 2009 174 228 238 10.2353/ajpath.2009.080764 19074615 PMC2631335

[B32] Yu X Wadghiri YZ Sanes DH Turnbull DH In vivo auditory brain mapping in mice with Mn-enhanced MRI Nat Neurosci 2005 8 961 968 10.1038/nn1477 15924136 PMC2034206

[B33] Kimura T Yamashita S Fukuda T Park JM Murayama M Mizoroki T Yoshiike Y Sahara N Takashima A Hyperphosphorylated tau in parahippocampal cortex impairs place learning in aged mice expressing wild-type human tau EMBO J 2007 26 5143 5152 10.1038/sj.emboj.7601917 18007595 PMC2140104

[B34] Rabin O Hegedus L Bourre JM Smith QR Rapid brain uptake of manganese(II) across the blood–brain barrier J Neurochem 1993 61 509 517 7687654 10.1111/j.1471-4159.1993.tb02153.x

[B35] Sloot WN Gramsbergen JB Axonal transport of manganese and its relevance to selective neurotoxicity in the rat basal ganglia Brain Res 1994 657 124 132 10.1016/0006-8993(94)90959-8 7820609

[B36] Liu CH D’Arceuil HE de Crespigny AJ Direct CSF injection of MnCl(2) for dynamic manganese-enhanced MRI Magn Reson Med 2004 51 978 987 10.1002/mrm.20047 15122680

[B37] Cross DJ Minoshima S Anzai Y Flexman JA Keogh BP Kim Y Maravilla KR Statistical mapping of functional olfactory connections of the rat brain in vivo NeuroImage 2004 23 1326 1335 10.1016/j.neuroimage.2004.07.038 15589097

[B38] Smith SM Flexible filter neighbourhood designation Proc 13th Int Conf on Pattern Recognition 1996 1 206 212

[B39] Jenkinson M Bannister P Brady M Smith S Improved optimization for the robust and accurate linear registration and motion correction of brain images NeuroImage 2002 17 825 841 10.1006/nimg.2002.1132 12377157

[B40] Chen XJ Kovacevic N Lobaugh NJ Sled JG Henkelman RM Henderson JT Neuroanatomical differences between mouse strains as shown by high-resolution 3D MRI NeuroImage 2006 29 99 105 10.1016/j.neuroimage.2005.07.008 16084741

[B41] Paxinos G Franklin KBJ The mouse brain: in stereotaxic coordinates 2001 2nd San Diego: Academic Press

